# Fluoride effect indicators in *Phaseolus vulgaris* seeds and seedlings

**DOI:** 10.7717/peerj.13434

**Published:** 2022-05-17

**Authors:** Ingrid Maressa Hungria de Lima e Silva, Arthur Almeida Rodrigues, Juliana de Fátima Sales, Douglas Almeida Rodrigues, Sebastião Carvalho Vasconcelos Filho, Cássia Lino Rodrigues, Priscila Ferreira Batista, Alan Carlos Costa, Marisa Domingos, Caroline Müller, Adinan Alves da Silva

**Affiliations:** 1 Laboratory of Seeds, Goiano Federal Institute of Science and Technology, Rio Verde, GO, Brasil; 2 Laboratory of Plant Anatomy, Goiano Federal Institute of Education, Rio Verde, GO, Brasil; 3 Laboratory of Ecophysiology and Plant Productivity, Goiano Federal Institute of Education, Rio Verde, GO, Brasil; 4 Núcleo de Pesquisa em Ecologia, Instituto de Botânica, São Pualo, SP, Brasil

**Keywords:** Bean, Chlorophyll a seedling fluorescence, Potassium fluoride, Morphoanatomical, Water quality

## Abstract

**Background:**

Fluoride (F) is one of the main environmental pollutants, and high concentrations are commonly detected in the air and in both surface and groundwater. However, the effects of this pollutant on seed germination and on the initial growth of crop seedlings are still poorly understood. In this context, the aim of this study was to assess morphoanatomical, physiological and biochemical fluoride effect indicators in *Phaseolus vulgaris* L. seeds and seedlings.

**Methods:**

*P. vulgaris* seeds were exposed to a liquid potassium fluoride solution (KF, pH 6.0) at concentrations of 0 (control), 10, 20, 30 mg L^−1^ for 7 days. A completely randomized experimental design was applied, consisting of four treatments with four replications each. During the experimental period, physiological (7 days) anatomical and histochemical (2 days), biochemical and chemical (4 days) assessments. An analysis of variance was performed followed by Dunnett’s test. to determine significant differences between the KF-exposed groups and control seeds; and a multivariate analysis was performed.

**Results:**

The germination parameters, and anatomical, morphological, physiological, biochemical and nutritional characteristics of the seedlings did not show negative effects from exposure to KF at the lowest doses evaluated. On the other hand, treatment with the highest dose of KF (30 mg L^−1^) resulted in a lower germination rate index and increase in abnormal seedlings, and higher electrical conductivity. A lower root length, magnesium content and photochemical efficiency were also observed. The exposure of *P. vulgaris* to KF, regardless the dose did not affect seeds anatomy and the accumulation of starch and proteins, in relation to the control group.

**Conclusions:**

Our findings demonstrated that *P. vulgaris* seedlings were tolerant to KF solutions up to 20 mg L^−1^, and sensitive when exposed to 30 mg KF L^−1^.

## Introduction

Environmental pollution is a universal issue, with increasing water scarcity and air and soil pollution observed worldwide (Wu et al., 2018). Fluoride (F) is one of the most phytotoxic contaminants released into the environment, usually at atmospheric precipitation concentrations ranging between 1 and 1,000 µg L^−1^, reaching values up to 10 mg L^−1^ in areas close to aluminum steel mills, coal burning and phosphate fertilizer factories (Smith & Hodge, 1979; Ding et al., 2013; Panda, 2015; Choubisa & Choubisa, 2016).

All forms of F pollution are considered serious and can severely affect plant production and human health (Walna et al., 2014; Kumari & Khan, 2018). According to Frazão et al. (2011), excess F ingestion is harmful, with the maximum human consumption fluorine limit established as 1.5 mg L^−1^. Eating or drinking F in excessive amounts causes fluorosis, a disease that occurs during the tooth formation period, resulting in opposite effects to the observed benefits of F at reduced doses. In many cases, visual F excess symptoms do not appear after unknown and prolonged exposure, resulting in health damage in those affected. Fluoride concentrations in water sources used in plant irrigation range from 1.5 to 5.0 mg L^−1^ worldwide (Abiye, Bybee & Leshomo, 2018), and in agricultural soils from 100 to 5,300 mg kg^−1^ (Rahman et al., 2018; Mikkonen et al., 2018).

Several crops require agricultural irrigation employing either surface or underground water, which is vital for global food production, comprising a strategy to ensure food security (Liang et al., 2016). The common bean (*Phaseolus vulgaris* L.) is among crops produced in irrigation systems (Justino et al., 2019). The bean plant is an important source of proteins, fibers, vitamins and minerals for human nutrition (Celmeli et al., 2018), being grown worldwide, with an estimated production of 29 million tons in 2019 (FAO, 2019). This crop is commonly irrigated with water from water bodies and artesian wells, which may contain high F concentrations. However, scarce information is available regarding F accumulation and its effects on bean growth and development.

Like most air pollutants, F penetrates plants through their stomata and cuticles (Sant’Anna-Santos et al., 2014), causing visual leaf changes and parenchymal cell damage under severe stress (Sharma & Kaur, 2018). Furthermore, F also alters the primary plant metabolism, compromises the respiration (Applegate & Adams, 1960), photosynthetic apparatus and causes oxidative stress in exposed plants even at the initial growth (Smith, 1962; Arndt, Flores & Weinstein, 1995; Rodrigues et al., 2018a). Fluoride effects on plants have been assessed in several studies. For example, drastic leaf blade changes have been reported for *Spondias dulcis* Forst., resulting in extensive necrotic areas, and damage to the entire anatomical structure (Silva et al., 2000). Visible and structural leaf damage have been reported for *Spondias purpurea* L. (Anjos et al., 2018), while reduced photosynthesis and increased respiration have been noted for *Byrsonima basiloba* A. Juss. (Rodrigues et al., 2018b). Both visual and anatomical changes in *Ricinus communis* L. (Rodrigues et al., 2020), and morphological and physiological modifications in *Sapindus saponaria* L. (Rodrigues et al., 2018a) have also been observed.

Given this scenario, studies regarding the effects of F accumulation on the physiology of seeds and plants with economic and food importance are paramount to highlight the importance of evaluating F contents in water employed in crop irrigation. Thus, the aim of the present study was to determine morphoanatomical, physiological F effect indicators in *P. vulgaris* seeds and seedlings.

## Materials & Methods

### Plant material and experimental design

Seeds of the super-early cycle carioca bean (*P. vulgaris*) cultivar BRS FC104 were used in all experiments. This cultivar is widely cultivated in Brazil and indicated for cultivation in irrigation systems. The experiments were carried out in a completely randomized design, consisting of four treatments: 0 (control), 10, 20 and 30 mg L^−1^ of potassium fluoride (KF), with four replicates.

### Germination test

Fifty *P. vulgaris* seeds by replicate were placed on two germitest sheets moistened with 2.5 times their dry weight with a liquid solution of KF (pH 6.0) at 0 (control), 10, 20 and 30 mg L^−1^ in order to simulate F concentrations found in irrigation water both in Brazil and worldwide (Abiye, Bybee & Leshomo, 2018; Martins et al., 2018). Then, the seeds were covered with a third germitest sheet already moistened with treatment solutions, rolled up, packed in transparent plastic bags, and maintained in a B.O.D incubator at a constant temperature of 25 °C (±0.5 °C), as described in the Rules for Seed Analysis (2009), for 7 days. Germination was evaluated daily, and the seeds were considered germinated where there was a two mm root protrusion to determine the total germination (TG, %). The germination rate index (GRI) was determined as described by Maguire (1962) as:

\begin{eqnarray*}\mathrm{GRI}=\sum _{i=1}^{n} \left( \frac{ni}{i} \right) \end{eqnarray*}

where:

*ni* = number of seeds germinated on day *i*;

*i* = number of days.

The stem and root length (cm), and stem diameter of the plants were measured in fourteen seedlings randomly collected per replicate using a digital caliper, 7 days after the beginning of the experiments. In addition, the seedlings were classified as normal, abnormal or dead.

### Electrical conductivity test

Seeds cell membrane stability was measured using the electrical conductivity of 50 seeds per replicate. The seeds previously weighed using an analytical balance were immersed in 75 mL of each KF solution (0, 10, 20 and 30 mg L^−1^) and maintained in a B.O.D incubator at 25 °C. After 24 h, the electrical conductivity of each solution was determined using a conductivity meter (Tec-4MP, Tecnal, Piracicaba, Brazil). The obtained values were divided by the initial sample weight and expressed as µS cm^−1^ g^−1^ (Krzyzanowski et al., 2020).

### Tetrazolium test

The seeds viability and vigor were performed by the tetrazolium test using 50 seeds for each replicate. The samples were pre-moistened in germitest paper with 2.5 times the dry seed matter with KF solutions (0, 10, 20 and 30 mg L^−1^) in plastic bags to prevent dehydration, at 25 °C, for 16 h. Subsequently, the seeds were totally immersed in a 0.075% tetrazolium solution in plastic cups and maintained in the dark, followed by heating in an oven at 40 °C for 150 min (Krzyzanowski et al., 2020). After staining, the samples were washed with water and seed vigor was evaluated using a table magnifer lamp (RT301 model; Ritek Electronics Co., China). Each seed was classified according to its types of damage (mechanical, moisture and bed bug damage), and damage location as: highest vigor (class 1), high vigor (class 2), medium vigor (class 3), low vigor (class 4), very low vigor (class 5), unviable (class 6) and dead (class 8), according to Krzyzanowski et al. (2020). Vigor seeds data were obtained by the sum of seeds categorized as classes 1, 2 and 3.

### Seed X-ray test

The X-ray test is a non-destructive test that allows checking the presence of internal damage to the seeds caused by insects or mechanical damage. Sixty-four seeds per replicate were immersed in KF solutions (0, 10, 20, and 30 mg L^−1^) for 48 h. After this time, the seeds were placed on transparent acrylic plates on double-sided adhesive tape and exposed to radiation using a Faxitron HPX-ray system (43855A model, Faxitron X-ray Corp, Wheeling, USA) at an intensity of 30 Kv for 15 s. Digital images were obtained and qualitatively analyzed to assess internal seed morphology.

### Morphoanatomical and histochemical characterization

Seed material was collected from the endosperm region 48 h after the seeds be immersed in KF solution (0, 10, 20 and 30 mg L^−1^), and fixed in Karnovsky’s solution (1965), for 24 h. For morphoanatomical analyses, the seed material was prepared as described by Rodrigues et al. (2020). Transverse sections were stained with toluidine blue-polychromatic (0.05%) in phosphate buffer (0,1 M, pH 6,8) (O’Brien, Feder & McCully, 1964). For histochemical analyses, sample sections were stained with lugol 10 g L^−1^ (Jensen, 1962) and Xylidine Ponceau (XP) for starch and total protein determinations, respectively (O’Brien & McCully, 1981). Qualitative morphoanatomical and histochemical observations were performed according to Rodrigues et al. (2020).

### Malonaldehyde (MDA) and hydrogen peroxide (H_2_O_2_) concentration

The level of lipid peroxidation was measured by quantifying MDA concentration according to Cakmak & Horst (1991). Seed samples (0.1 g) obtained after 4 days of the germination test were homogenized in 10 mL of trichloroacetic acid (TCA; 0.1% w/v), and centrifuged at 15,000 g, at 4 °C, for 10 min. The supernatant (one mL) was mixed with four mL of thiobarbituric acid solution (TBA; 0.5% w/v of TBA in 20% TCA). The reaction mixture was heated at 95 °C, for 30 min in a water bath. The reaction was stopped in a subsequent ice bath. The samples were centrifuged at 10,000 g, at 4 °C, for 10 min, and the supernatant absorbances were determined at 532 and 600 nm (Heath & Packer, 1968) using a UV-visible spectrophotometer (Evolution 60S model; Thermo Fisher Scientific Inc., MA, EUA). The concentration of MDA was calculated using the molar extinction coefficient of 155 mM^−1^ cm^−1^ (Heath & Packer, 1968) according to the following equation: MDA (nmol mL^−1^) = [(*Abs*_532_ −*Abs*_600_)/155,000] × 10^6^, and expressed as nmol MDA g^−1^ fresh weight.

The production of reactive oxygen species was estimated by the H_2_O_2_ concentration also after 4 days of germination in the F solutions. Seeds samples (0,2 g) were homogenized in liquid nitrogen and then in two mL of potassium phosphate buffer (50 mM, pH 6.5, containing 1 mM hydroxylamine). After filtration, the homogenate was centrifuged at 10,000 g, at 4 °C, for 15 min (Kuo & Kao, 2003). Supernatant Aliquots (50 µL) of the supernatant were added to a reaction medium consisting of ammoniacal ferrous sulfate FeNH_4_(SO_4_) (100 µM), sulfuric acid (25 mM), xylenol orange (250 µM) and sorbitol (100 mM), in a final volume of two mL (Gay & Gebicki, 2000). After 30 min in the dark, the absorbance of the samples was determined at 560 nm in a UV–VIS spectrophotometer. In parallel, a blank sample were obtained for each sample and subtracted from sample absorbance values. The H_2_O_2_ concentration was estimated based on an H_2_O_2_ standard curve, and expressed as µmol g^−1^ fresh weight.

### Fluoride and macronutrients content

Four days after germination teste, F and macronutrients content were analysed. Fluoride determinations was carried out in previously dried and ground seeds soaked with different KF concentrations. Fluoride determination was performed according to Silva et al. (2020).

For macronutrients analyses, seed samples were washed in distilled water and dried in paper bags at 60 °C under induced air circulation conditions until constant mass. The material was ground in a Willey type mill (20-mesh sieve), ashed in a muffle and the minerals extracted by nitroperchloric digestion according to Embrapa (2009). The nutrients calcium (Ca) and magnesium (Mg) were analyzed by atomic absorption spectrophotometry, and potassium (K) using a flame photometry.

### Chlorophyll *a* seedling fluorescence

Photosynthetic efficiency and physiological changes were assessed by the chlorophyll a fluorescence measurement. Variables of chlorophyll fluorescence were measured 7 days after KF solution (0, 10, 20, and 30 mg L^−1^) on the germination test using a modulated Imaging-PAM fluorometer (Maxi version; Heinz Walz GmbH, Effeltrich, Germany). Image capture and equipment calibration were performed according to Lima et al. (2017); from these, the maximum photosystem II (PSII) quantum yield was calculated [F_v_/F_m_ = (F_m_−F_0_)/F_m_] (Genty, Briantais & Baker, 1989). After sample illumination, saturation pulses were applied to determine the initial fluorescence (F), and the maximum fluorescence (F_m′_) in light-acclimated leaves. These variables were used to estimate the apparent electron transport rate [ETR = (F_m′_ −F)/F_m′_ × PAR × LeafABS × 0.5] (Bilger, Schreiber & Bock, 1995), where PAR is the photon flux (µmol m^−2^ s^−1^) in the leaves, LeafABS is the fraction of incident light absorbed by the leaves, and 0.5 is the fraction of excitation energy directed to the PSII (Laisk & Loreto, 1996). The quenching of regulated non-photochemical dissipation [Y_NPQ_ = (F_s_/F_m′_) − (F/F_m_)] and the quenching of non-regulated non-photochemical dissipation [Y_NO_ = F_s_/F_m_] were calculated according to Genty, Briantais & Baker (1989) and to Hendrickson, Furbank & Chow (2004).

### Statistical analyses

The obtained data were submitted to previous analyzes of homogeneity (Levene test) and normality (Shapiro–Wilk test) of the error. Following data normality confirmation, ANOVA was performed, and the treatment means were compared to the control using the Dunnett test, considering 5% (*) and 1% (**) of probability. All analyses were performed using R software version 3.1.2 (R Core Team, 2021). For multivariate analysis, data were initially standardized by square root transformation and scaled by mean centering. Principal component analysis (PCA) and score plots were obtained using the MetaboAnalyst platform version 5.0 (https://www.metaboanalyst.ca).

## Results

### Germination parameters and electrical conductivity

*P. vulgaris* seeds did not show statistical differences in total germination (∼93%) when exposed to different KF solutions, compared to the control (Table 1). Seeds vigor level and viability were not affected by KF treatments. However, germination rate index reduced (10%) and electrical conductivity increased (17%) when the seeds were exposed to the highest KF doses (30 mg L^−1^) when compared to the control (Table 1). Also, a reduction in normal seedlings (7%) and an increase in abnormal seedling (33%) was observed at the highest KF dose (Table 2). Dead seedling was not altered by KF treatments.

**Table 1:** Germination parameters and electrical conductivity. Total germination (%), vigor level (%), seeds viability (%), germination rate index (GRI), and electrical conductivity (EC) in *Phaseolus vulgaris* L. seeds exposed to 0, 10, 20 and 30 mg L^−1^ of potassium fluoride (KF).

**KF** **(mg L^−1^)**	**TG** **(%)**	**Vigor level (%)**	**Viability (%)**	**GRI**	**EC** **(µS cm^−1^ g^−1^)**
0	94 ± 1.41	88 ± 2.06	90 ± 2.00	29 ± 0.55	113 ± 2.60
10	93 ± 1.26	88 ± 1.83	94 ± 2.71	29 ± 0.76	115 ± 4.07
20	93 ± 2.75	85 ± 2.08	90 ± 1.41	31 ± 0.30	116 ± 4.55
30	93 ± 1.29	82 ± 0.82	87 ± 1.26	26^**^±0.35	132^*^±3.39
**One-Way ANOVA**					
*F* (*t*-test)	0.1558	2.4040	2.5196	12.8859^**^	5.3415^*^
*P*	0.9239	0.1183	0.1074	0.0004	0.0143

**Notes.**

Data represent mean ± SEM (*n* = 4).

Asterisks indicate significant differences at 5% (*) of probability, between KF and control treatments, according to the Dunnett test.

**Table 2:** X-ray and tetrazolium seed analyses, and initial growth. Percentage of normal, abnormal and dead seeds, and initial growth of root length (RL), and stem length (SL) in *Phaseolus vulgaris* L. seeds exposed to 0, 10, 20 and 30 mg L^−1^ of potassium fluoride (KF).

**KF** **(mg L^−1^)**	**Normal seedlings** **(%)**	**Abnormal seedlings** **(%)**	**Dead seedlings** **(%)**	**RL** **(cm)**	**SL** **(cm)**
0	44 ± 0.85	4 ± 0.65	3 ± 0.75	6.7 ± 0.14	4.0 ± 0.12
10	43 ± 0.71	4 ± 0.25	3 ± 0.48	6.0^**^±0.13	3.7 ± 0.13
20	42 ± 0.29	5 ± 0.41	4 ± 0.50	6.6^**^±0.17	3.8 ± 0.15
30	41^*^± 0.41	6^*^± 0.75	3 ± 0.48	5.7^**^±0.14	3.8 ± 0.15
**One-Way ANOVA**					
F (*t*-test)	4.4366	5.3103	0.4426	8.7295	0.9567
*p*	0.0256	0.0146	0.7269	<.0001	0.4177

**Notes.**

Data represent mean ± SEM (*n* = 4).

Asterisks indicate significant differences at 5% (*) and 1% (**) of probability, between KF and control treatments, according to the Dunnett test.

### X-ray and tetrazolium seed analyses, and initial growth

The X-ray seed images and tetrazolium test revealed ideal morphological *P. vulgaris* structures and healthy cotyledons and endosperm, with no morphological damage with increasing KF concentrations (Fig. S1, Supplementary Material). However, root length was reduced between 10% and 15%, compared to the control, when exposed to the lowest (10 mg L^−1^) and the highest (30 mg L^−1^) KF solutions (Table 2). The stem length was not significantly affected by KF treatments (Table 2).

### Anatomical and histochemical seed characterizations

**Figure 1:**
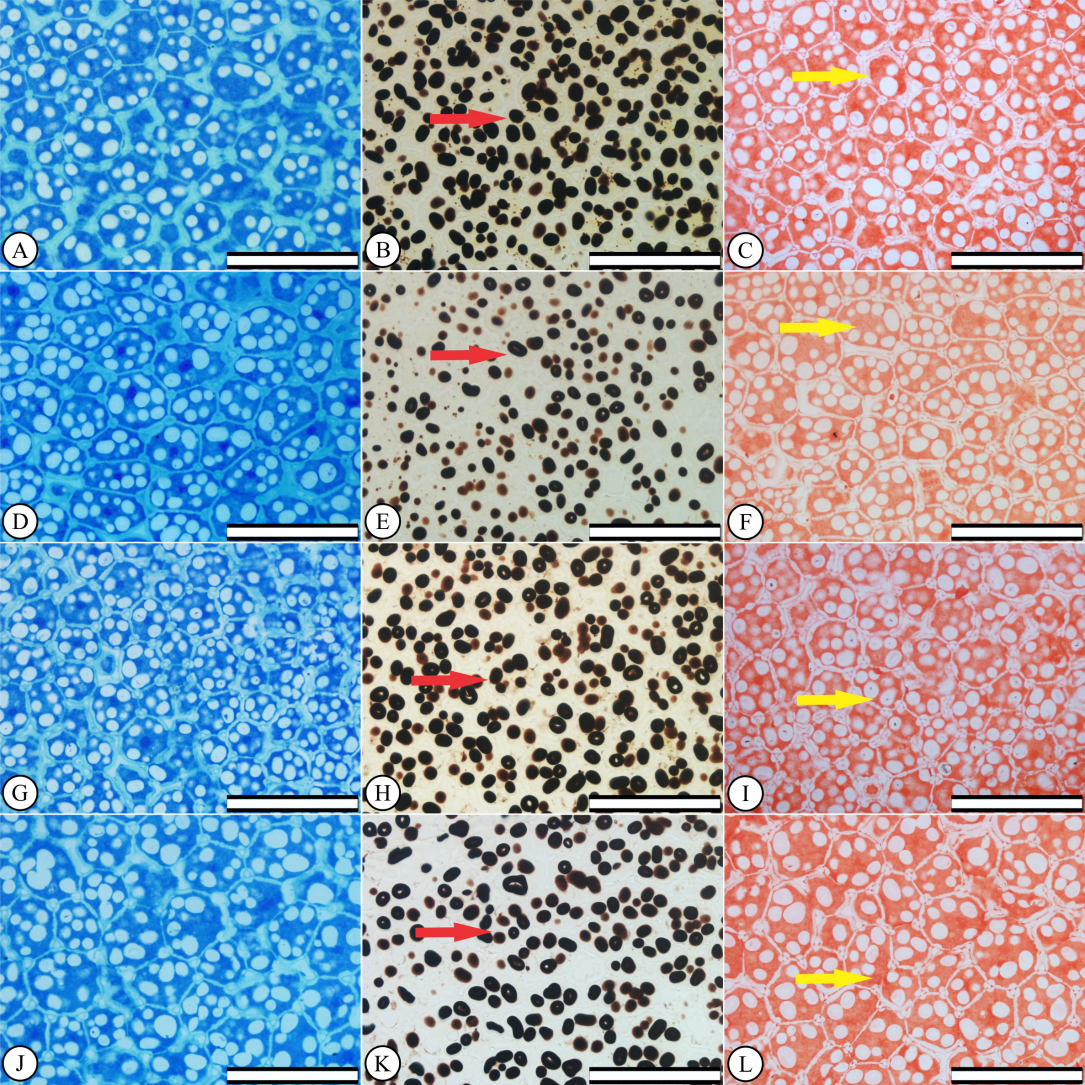
Anatomical and histochemical seed characterizations. *Phaseolus vulgaris* seed endosperms after the germination test. (A–C) control, (D–F) 10 mg KF L^−1^KF, (G–I) 20 mg KF L^−1^ and (J–L) 30 mg KF L^−1^. Scale bar = 100 µm. Left column: anatomy characterization. Middle column: arrows indicate starch accumulation. Right column: arrows indicate protein accumulation.

Increasing KF concentrations did not caused cell changes in *P. vulgaris* endosperms (Figs. 1D, 1G and 1J), compared to the control treatment (Fig. 1A). Starch accumulation, *i.e.,* large areas stained in black by lugol, were observed both in the control (Fig. 1B) and the KF-exposed seeds (Figs. 1E, 1H and 1K), showing no qualitative differences between treatments. Protein-stained areas also indicate that no protein leakage from the endosperm cell occurred after KF treatments (Figs. 1C, 1F, 1I and 1L).

### MDA and H_2_O_2_ contents

KF exposure did not affect the concentration of MDA and H_2_O_2_ in *P. vulgaris* seedlings (Fig. S2).

### Seed fluoride and macronutrient contents

F content increased in *P. vulgaris* seeds in 74% and 85% when exposed to 20 and 30 mg L^−1^ KF, respectively, when compared to the control treatment (Table 3). Calcium (Ca) and potassium (K) seed contents were not significantly altered by KF treatments (Table 3). Mg content decreased by 10% at the highest KF concentration (30 mg L^−1^), when compared to the control (Table 3).

**Table 3:** Seed fluoride and macronutrient contents. Fluoride (F), calcium (Ca), potassium (K) and magnesium (Mg) contents in *Phaseolus vulgaris* L. seeds exposed to 0, 10, 20 and 30 mg L^−1^ of potassium fluoride (KF).

**KF** **(mg L^−1^)**	**F** **(g Kg^−1^)**	**Ca** **(g Kg^−1^)**	**K** **(g Kg^−1^)**	**Mg** **(g Kg^−1^)**
0	0.26 ± 0.022	2.33 ± 0.25	14.10 ± 0.06	2.13 ± 0.10
10	0.36 ± 0.024	2.27 ± 0.07	14.33 ± 0.12	2.20 ± 0.07
20	0.45^**^±0.029	2.05 ± 0.13	14.20 ± 0.08	2.20 ± 0.04
30	0.48^**^±0.043	2.02 ± 0.20	14.45 ± 0.15	1.91^*^±0.04
**One-Way ANOVA**				
F (*t*-test)	10.3285	0.7617	1.9480	3.8665
*P*	0.0011	0.5369	0.1758	0.038

**Notes.**

Data represent mean ± SEM (*n* = 4).

Asterisks indicate significant differences at 5% (*) and 1% (**) of probability, between KF and control treatments, according to Dunnett test.

### Chlorophyll *a* fluorescence in *P. vulgaris* seedlings

**Figure 2:**
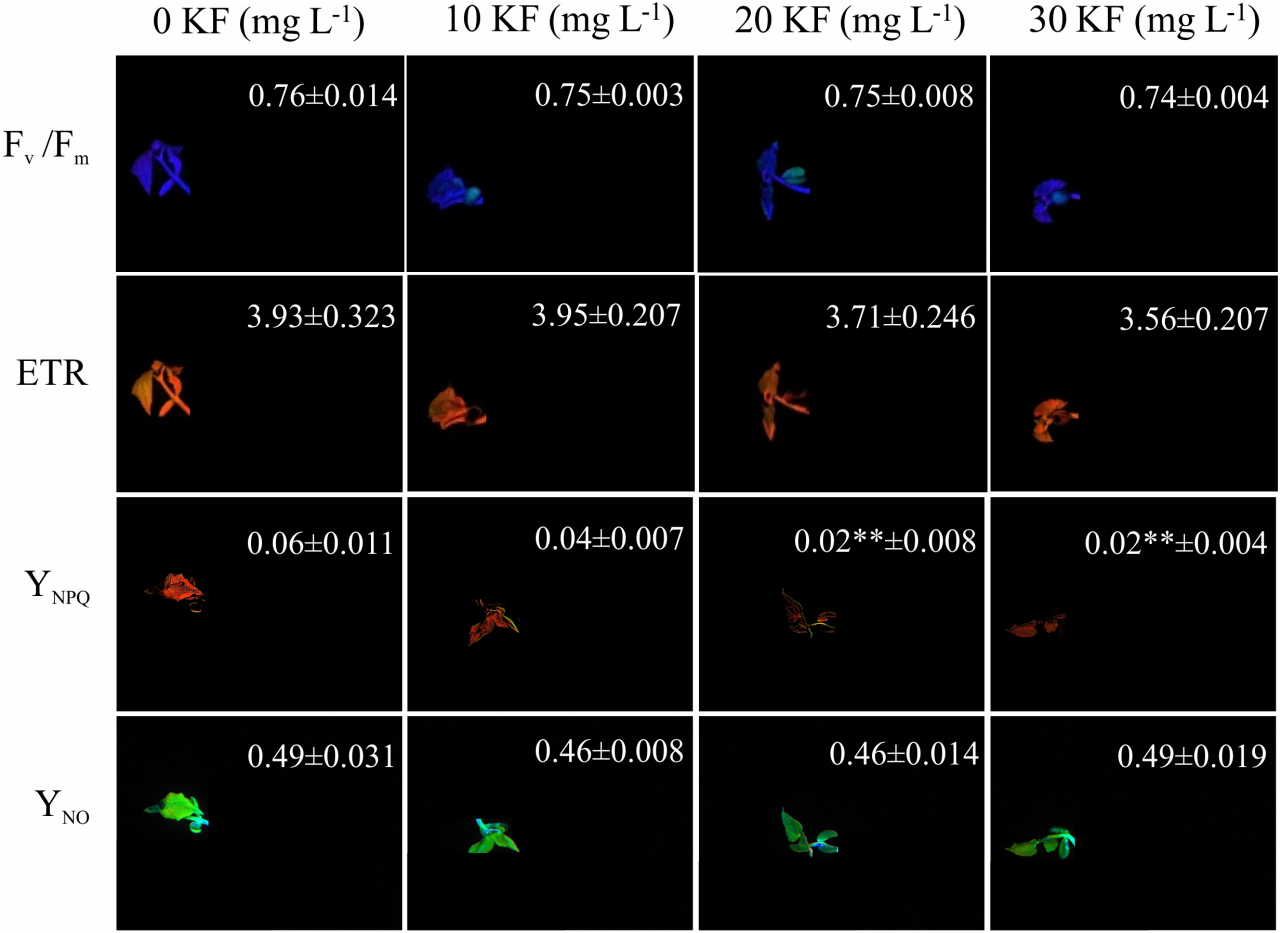
Chlorophyll *a* fluorescence. Potential quantum yield of photosystem II (*F*_*v*_/*F*_*m*_), electron transport rate (ETR), regulated non-photochemical quenching (NPQ) and non-regulated non-photochemical quenching (qN) in *Phaseolus vulgaris* L. seeds exposed to 0, 10, 20 and 30 mg L^−1^ of potassium fluoride (KF). Data represent mean ± SEM (*n* = 4). Asterisks indicate significant differences at 5% (*) and 1% (**) probability, between KF and control treatments, according to the Dunnett test.

The potential quantum yield of photosystem II (*F*_*v*_/ *F*_*m*_) and the electron transport rate (ETR) showed a slight reduction with increasing the KF dose, but it was not this was not statistically significant (Fig. 2). Seed exposure to 30 mg L^−1^ KF resulted in a reduction in the quenching of regulated non-photochemical dissipation (Y_NPQ_) and did not affect the quenching of non-regulated non-photochemical dissipation (Y_NO_) in *P. vulgaris* seedlings (Fig. 2).

### Principal component analysis

**Figure 3:**
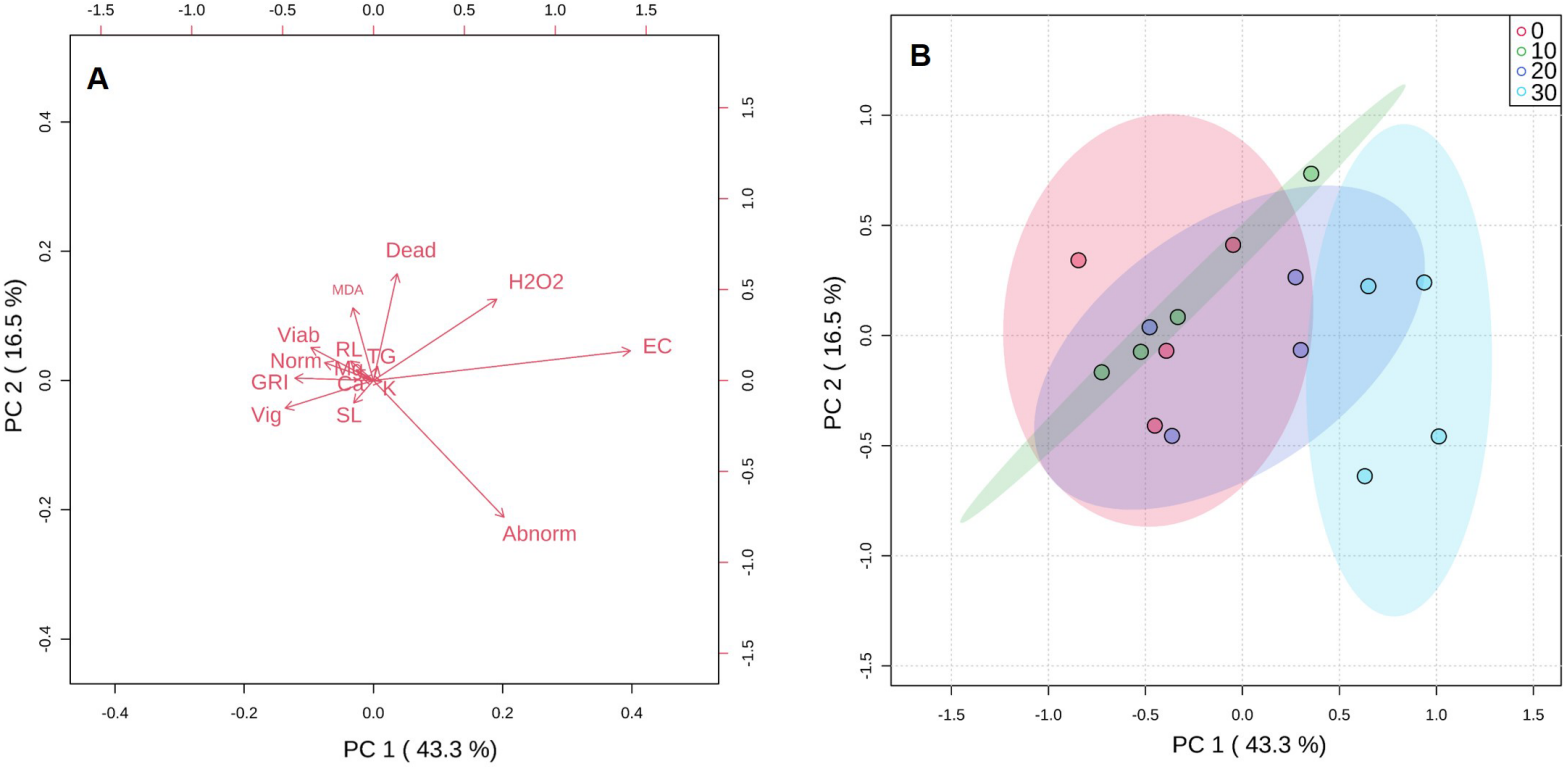
Principal component analysis (A) and score plot (B) for germination parameters, vigor, viability, initial growth, electrical conductivity, malonaldehyde and hydrogen peroxide concentrations, and macronutrients content of seeds exposed to 0, 10, 20 and 30 mg L^−1^ of potassium fluoride (KF).

Analysis of the first three principal components explained in total 74.6% of the total variation observed (Fig. 3). The greatest contribution to the first component (PC1) was observed by abnormal seeds, EC, GRI, and vigor. Dead and abnormal seeds, and also H_2_O_2_ contributed to PC2 (Fig. 3A). The score plot indicated that there was a high degree of overlap between 10 and 20 mg KF L^−1^ treatments. However, a clear separation between control treatment and the highest KF dose (30 mg L^−1^) was observed (Fig. 3B).

## Discussion

Seed germination is widely recognized as important for plant growth, and the effects of F toxicity in this stage are still poorly known (Stanley et al., 2002; Sabal, Khan & Saxena, 2006). Many plants are especially sensitive to F, which can affect the plant growth, cause chlorosis and necrosis in leaf, among others damages (Elloumi et al., 2005; Chatterjee et al., 2020). *P. vulgaris* seedlings maintained their vigor, viability, morphoanatomical, biochemical and nutritional characteristics at doses of 10 and 20 mg KF L^−1^. However, a decrease of *P. vulgaris* germination rate indices (Table 1) and root lengths (Table 2) were observed after exposure to the highest KF dose (30 mg L^−1^), as well as an increased membrane disorganization (Table 1). Membrane damage can also affect seedling metabolism as described for *Triticum aestivum* L. seeds exposed to lead (Lamhamdi et al., 2011), and *Zea mays* L. and *Solanum melongena* L. exposed to sodium fluoride (Ahmed et al., 2020; Ghaffar et al., 2020).

The seed vigor and internal and external morphological seed structures play an important role in the germination process and can be used to estimate the plant’s ability to establish itself, under different stress and climate conditions (Pereira et al., 2019). The vigor, viability of *P. vulgaris* seeds did not differ significantly among treatments, despite a slight effect on the treatment with the highest KF dose and negative effects on initial growth (Tables 1 and 2). In addition, X-ray analysis did not identify morphological seed alterations after 48 h of exposure to KF (Fig. S1). This indicates that both the tetrazolium test and X-ray analysis were not effective indicators of KF when performed early in the germination process of beans. This occurs since even with no morphological changes, the endosperm is capable of sensing environmental signals (Yan et al., 2014), which was observed by the increase in abnormal seeds (Table 2). Seed germination is an energy-dependent process (ATP) and many enzymes are involved in transforming complex reserve substances into simpler forms during this step, which are then translocated to seedling growth regions (Mondal, 2017). Therefore, higher F levels may interfere with enzymatic activities and energy release, and, consequently, changes in seeds metabolic activity (Datta et al., 2012), which would explain the changes observed in the initial growth of *P. vulgaris* under the highest KF dose.

Anatomical characteristics of the endosperm of *P. vulgaris* seeds were not affected by KF exposure at the early germination stage, regardless the doses (Fig. 1A). The seeds also maintained the accumulation of starch and proteins similar to the control seeds (Figs. 1B–1C). It is not surprising that in 24 h the seed reserves have not been altered. Seed hydration increases the embryo’s metabolism, and the breakdown of starch and proteins reserves will provide the mobilization of carbon and nitrogen for germination process and the initial development of seedlings tissues (Yan et al., 2014). In addition, the presence of total proteins does not guarantee seedling vigor. Ehrhardt-Brocardo, Coelho & Souza (2022) identified and quantified proteins present during the initial stage of germination of common bean plants. According to the authors, different proteins were observed between the genotypes with low and high vigor, and the protein lipoxygenase was observed only in the genotypes with high vigor.

Crops also have different responses to the F action. Fina et al. (2016) evaluating the effect of fluoride on the germination and growth of crops found that *Z. mays* and *Glycine max* (L.) Merr. were more sensitive to F than *Sorghum vulgare* Pers., mainly due to the lower vigor observed in *Z. mays* and *G. max* plants. Besides, a significant loss of seedling vigor can impair the growth and development of seedlings in the field.

The highest KF dose affected Ca and Mg nutrients in seedlings (Table 3). Ca and Mg were also reported to reduce after F application in *Amygdalus communis* L. seedlings (Elloumi et al., 2005). The reduction in these nutrients can impair the carbohydrate metabolism and inhibit amylase activity, which is essential for the seed germination (Gupta, Banerjee & Mondal, 2009; Ram, Verma & Gadi, 2014). Ca^2+^ ions, along with other factors such as chloride, calmodulin proteins, associated with membrane potential, participate in the regulation of F accumulation in plants. A putative channel for F also seems to be involved, but this transport mechanism is still not fully elucidated (Gadi et al., 2021). The complexation of F with cations as Ca and Mg is the main reason for the reduction in these ions (Gao et al., 2014; Banerjee & Roychoudhury, 2019). Ca and Mg deficiency can affect plant growth and development since they are important cofactors for activation of many enzymes of metabolic reactions and signaling pathways in plants (Banerjee & Roychoudhury, 2019).

The post-germination period is critical, as developing seedlings must reach an autotrophy state before the depletion of stored nutrients. Thus, the evaluation of parameters associated to seedling photosynthesis through chlorophyll fluorescence-based techniques becomes useful in the fast and objective identification of patterns linked to physiological seed potential (Ariyarathna, Weerasena & Beneragama, 2020; Sánchez-Moreiras et al., 2020; Vidak et al., 2020). The variables F_v_/F_m_ and ETR were slightly reduced in seedlings of *P. vulgaris* originated from seeds treated with the highest dose of KF (Fig. 2). This suggests an adverse effect of the pollutant on the photochemical efficiency of the seedlings and agrees with the verified by Verma et al. (2022), where F reduced the F_v_/F_m_ of *Pisum sativum* L. seedlings through damage to cell membranes and chlorophylls degradation. Fluoride has been reported to causes lipid peroxidation, which can be associated to the higher H_2_O_2_ production and other reactive oxygen species (ROS) (Yadu, Chandrakar & Sahu, 2016; Sharma et al., 2019). The ROS can be intensified by environmental stressors, and they affect membrane phospholipids, resulting in damage to intracellular organelles (Zhang et al., 2021).

Since photochemistry is reduced in plants under stress, it is expected that forms of non-photochemical dissipation will increase as a plant protection mechanism. The Y_NPQ_ is related to the thermic dissipation by the xanthophyll cycle, which occurs through the formation of a pH range (ΔpH) in the chloroplast lumen in a xanthophyll-regulated mechanism (Golding & Johnson, 2003; Joliot & Joliot, 2006), and Y_NO_ increases suggest the occurrence of photodamage in the energy transfer to Qa (Huang, Zhang & Cao, 2010). Thus, the increase of both Y_NPQ_ and Y_NO_ act as a way of protecting the photosynthetic apparatus of plants when PSII function is impaired (Murchie & Ruban, 2020). However, in the opposite way, the seedlings of *P. vulgaris* in this study showed a decrease in Y_NPQ_, and Y_NO_ was not altered (Fig. 2). Accumulation of high levels of F was reported to reduce xanthophyll concentrations in leaves of adult *Carthamus tinctorius* L. plants (Ghassemi-Golezani & Farhangi-Abriz, 2019). Similar response may have occurred in the seedlings of *P. vulgaris*, which would explain the reduction in energy dissipation as heat, expressed by the Y_NPQ_. In addition, the kinetics of xanthophyll cycle enzymes are regulated by membrane fluidity (Latowski et al., 2002). Thus, damage to cell membranes caused by F was identified as the cause of the reduction of pigments associated with photosynthesis and the decrease in the photochemical efficiency of PSII (Ghassemi-Golezani & Farhangi-Abriz, 2019). Within this context, the toxicity of F on *P. vulgaris* seeds denotes a factor that compromises the photosynthetic capacity of seedlings. Thus, this can reduce the physiological performance of plants originating from seeds exposed to the pollutant.

## Conclusions

*P. vulgaris* seeds demonstrate KF tolerance as revealed by anatomical, biochemical, histochemical and physiological characteristics maintenance when exposed up to 20 mg KF L^−1^. At the highest KF dose (30 mg L^−1^), a reduction in GRI, root length, Mg content and photochemical efficiency, and an increase in electrical conductivity and abnormal seedlings indicated the sensitivity of *P. vulgaris* to high doses of KF. Thus, irrigation water assessments and F determinations in seeds and plants are paramount in order to reduce potential public health problems.

## Supplemental Information

10.7717/peerj.13434/supp-1 Figure S1 X-ray seed analyses Internal seed morphology in *Phaseolus vulgaris* L. seeds exposed to (A) 0 mg L^−1^ (control), (B) 10 mg L^−1^, (C) 20 mg L^−1^, and (D) 30 mg L^−1^ of potassium fluoride (KF). Scale bar: 2 cm.

10.7717/peerj.13434/supp-2 Figure S2 MDA and H2O2 contents Malonaldehyde (MDA) and hydrogen peroxide (H2O2) concentrations in *Phaseolus vulgaris* L. seeds exposed to 0, 10, 20 and 30 mg L^−1^ of potassium fluoride (KF).

10.7717/peerj.13434/supp-3 Data S1 Raw data Germination parameter, morphological, nutritional and physiological raw data of *Phaseolus vulgaris* L. seeds exposed to 0, 10, 20 and 30 mg L^−1^ of potassium fluoride (KF).

## Additional Information and Declarations

### Data Availability

The following information was supplied regarding data availability: The raw measurements are provided in the Supplementary File.
