# Supplementary figures and images for: Fluoride effect indicators in Phaseolus vulgaris seeds and seedlings
Source: PeerJ. 2022 May 17;10:e13434. doi: 10.7717/peerj.13434 (PMC9121868; doi:10.7717/peerj.13434)

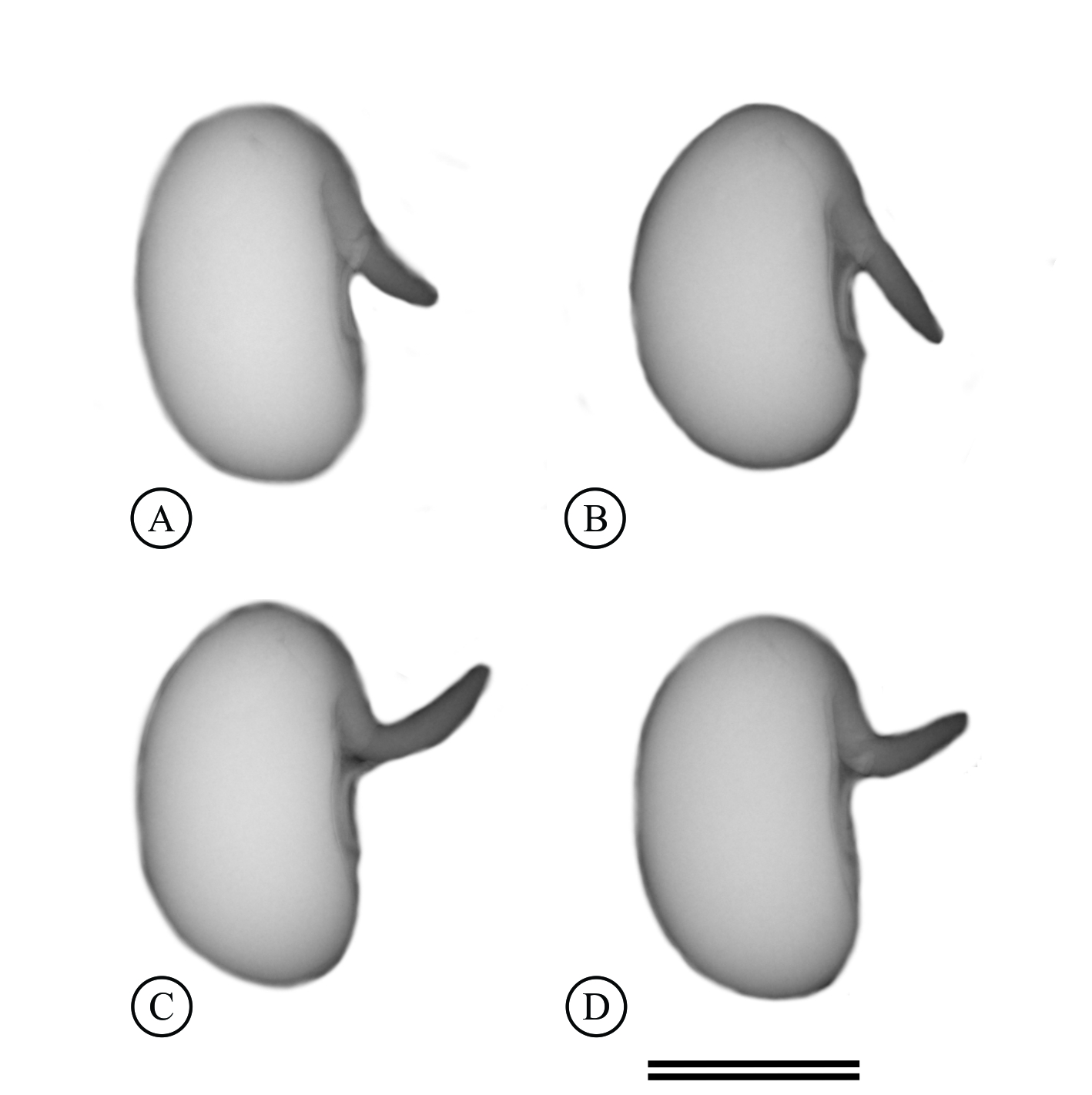

Supplement: Figure S1 — Internal seed morphology in Phaseolus vulgaris L. seeds exposed to (A) 0 mg L−1 (control), (B) 10 mg L−1, (C) 20 mg L−1, and (D) 30 mg L−1 of potassium fluoride (KF). Scale bar: 2 cm. [file peerj-10-13434-s001.jpg]

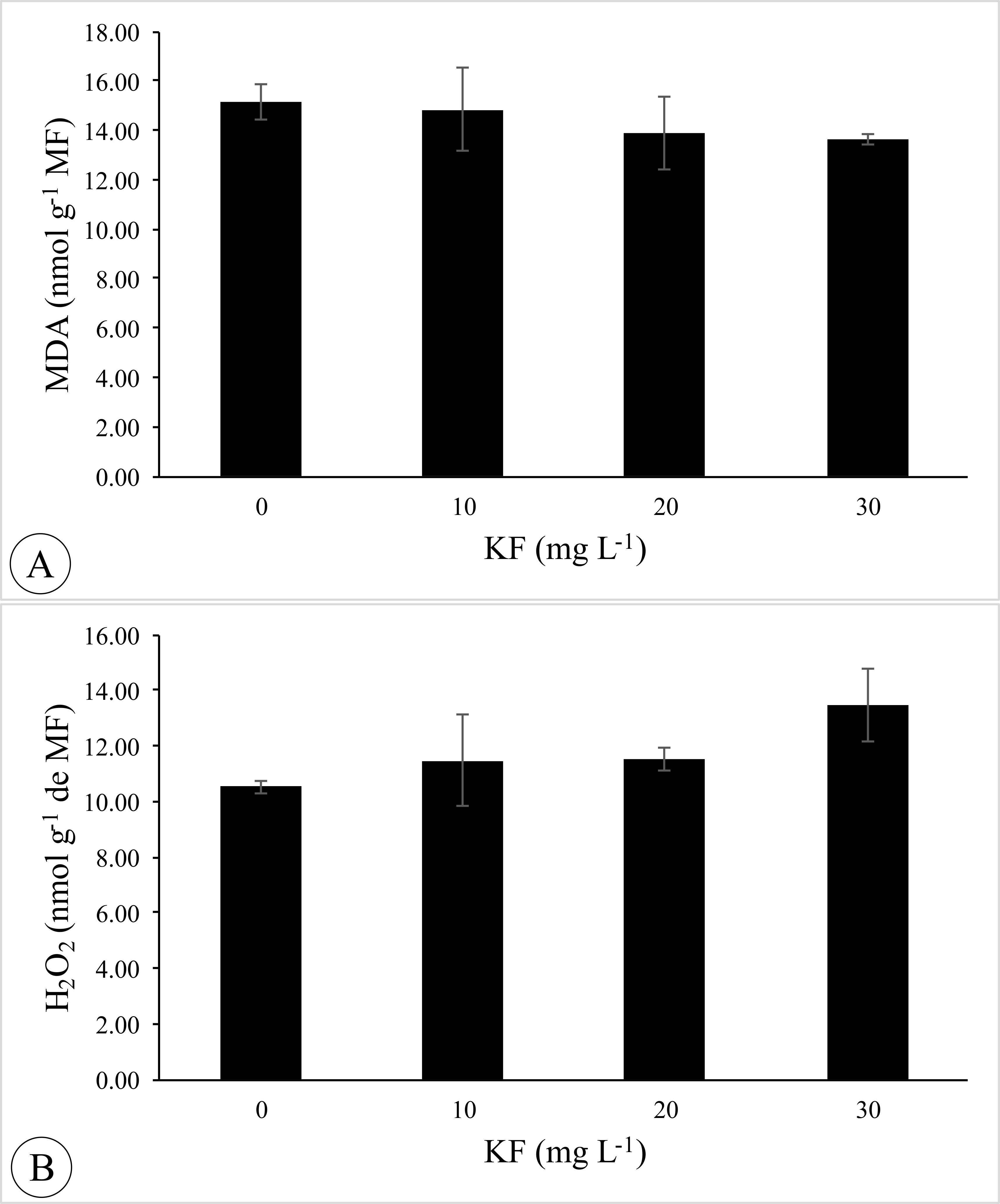

Supplement: Figure S2 — Malonaldehyde (MDA) and hydrogen peroxide (H2O2) concentrations in Phaseolus vulgaris L. seeds exposed to 0, 10, 20 and 30 mg L−1 of potassium fluoride (KF). [file peerj-10-13434-s002.jpg]
